# Guest Editorial

**DOI:** 10.1289/ehp.1003273

**Published:** 2011-01

**Authors:** Kalpana Balakrishnan, R.S. Dhaliwal, Bela Shah

**Affiliations:** Department of Environmental Health Engineering and Centre for Advanced Research in Environmental Health for the Indian Council of Medical Research, Sri Ramachandra University, Chennai, India, E-mail: kalpanasrmc@vsnl.com; Indian Council of Medical Research, New Delhi, India

Environmental and occupational risk factors contribute to nearly 40% of the national burden of disease in India [World Health Organization (WHO) 2002], with air pollution ranking among the leading risk factors. Despite the ubiquity of extreme exposure situations that may have substantial contributions from outdoor, indoor, and occupational microenvironments, health effects research in India has been sparse and compartmentalized, resulting in a limited pool of integrated evidence from local studies. Consequently, policy and regulatory standards have been driven largely by global evidence. Here, we present a rationale for developing integrated health research frameworks that jointly address outdoor and indoor exposures in rural and urban settings to generate representative impact data and inform development and regulatory efforts in India.

Levels of ambient air pollution uniformly exceed the recently revised WHO air quality guideline (AQG) levels (WHO 2006) across most cities in India, with almost 80 nonattainment cities and towns and 24 critically polluted hotspots identified by the Central Pollution Control Board (CPCB), Government of India (CPCB 2009). An estimated 120,600 deaths are attributed to outdoor air pollution each year in India (WHO 2002), but few Indian studies have informed this estimate, and recent reviews (Health Effects Institute 2004; Wong et al. 2008) indicated that most have been limited to cross-sectional studies of respiratory symptoms and lung function in relation to interzonal differences in air quality within cities, with only a few time-series studies of all-cause mortality conducted in selected populations. However, with routinely collected information becoming more accessible—including electronic data on daily particulate matter < 10 μm in aerodynamic diameter (PM_10_), sulfur dioxide, and nitrogen dioxide concentrations across 341 CPCB stations in 125 cities and towns and mortality/morbidity data from many municipalities and hospitals—multicity, multipollutant studies of short-term health effects are increasingly feasible. Although such studies alone will not provide the breadth of evidence needed to estimate the burden of chronic, as well as acute, health outcomes, it may be possible to extrapolate long-term risks based on short-term effects, which appear to be similar to those in other countries with more complete data.

According to the Indian National Census of 2001 (Office of The Registrar General & Census Commissioner, New Delhi, India), 75% of Indian households use solid fuels (primarily firewood and cow dung), including up to 90% of households in some rural areas. An estimated 400,000 deaths from acute lower respiratory infections (ALRI) in children < 5 years of age and 34,000 deaths from chronic obstructive pulmonary disease (COPD) in women are attributed annually to indoor air pollution (Smith 2000; Smith et al. 2004). Large data sets on indoor air quality measurements in solid fuel using households in India [International Agency for Research on Cancer (IARC) 2010] have been used to examine temporal, spatial, and multipollutant exposure patterns and to identify household level determinants and indicators of exposure (Balakrishnan et al. 2004). Data from rural indoor settings provide unequivocal evidence of extreme exposures that often are 15–30 times higher than WHO AQG recommendations. However, although several Indian studies have been included in systematic reviews of associations between exposure to solid fuel smoke and ALRI (Dherani et. 2008), low birth weight (Pope et al. 2010), and COPD (Kurmi et al. 2010), available quantitative exposure information has not been integrated into studies of health outcomes in India. In addition, limited evaluations of improved biomass stoves have shown that exposures still exceed WHO AQG guidelines and that the feasibility of sustained use is uncertain. Given the absence of information on exposure–response functions in relation to solid fuel smoke exposure and the economic impracticality of liquefied petroleum gas or electricity as near-term interventions, there is a critical need to augment efforts to estimate the avoidable burden of disease for multiple alternative interventions.

The nature of air pollution exposures in India presents unique challenges but also affords important opportunities for health effects research. For example, a significant portion of outdoor air pollution is due to household solid fuel smoke emissions in many rural areas, and exposures in urban areas are also likely to differ from settings in developed countries given the comparability of population and vehicular densities in residential, commercial, and industrial zones and the virtual absence of home air-conditioning. The urban outdoors is also the living environment for the poorest populations in India, and there is a growing periurban population that is poorly understood in terms of both exposures and health. Finally, without reliable job-exposure profiles, the nature and extent of exposure attributable to occupational settings is uncertain, and estimating exposure based only on measurements at urban ambient air quality monitors or on indoor solid fuel smoke exposures may result in significant underestimation.

As has been observed in other countries (Kan et al. 2009), evaluating combined exposures and effects of indoor and outdoor air pollution in longitudinal frameworks would *a*) reduce ambiguities in effect estimates; *b*) optimize health impact/climate cobenefit information for risk communication across rural and urban (or even developed and developing country) settings; *c*) add momentum for intervention efforts; *d*) increase efficiency of environmental epidemiology research; and *e*) jointly inform policy in the environment, health, and energy sectors. With emerging implications of emissions from both outdoor and indoor sources for climate change and health, such integration would also facilitate national efforts for emission reduction (Smith and Balakrishnan 2009).

In an effort to close existing data gaps, the Indian Council of Medical Research (ICMR) has established a Centre for Advanced Research in Environmental Health that will focus on air pollution and examine a range of exposures and outcomes in a rural–urban pregnant mother–child cohort and an adult endovascular disease cohort. Land-use regression modeling and select gene–environment related end points are also being examined in a nested subsample. In addition, the center will engage in capacity building to address human resource needs by developing training modules for different categories of professionals.

With high receptivity among research and funding agencies within and outside India and an increasing base of local research capacities in air pollution, India provides fertile grounds for global research partnerships that can facilitate the launch of strategic epidemiological studies to promote the timely application of international research and accelerate progress toward achieving universal access to clean air. ICMR initiatives to develop collaborative projects on interventions related to household solid fuel combustion under the ongoing Indo-US program on Environmental and Occupational Health reflect first steps toward achieving this goal.

## Figures and Tables

**Figure:**
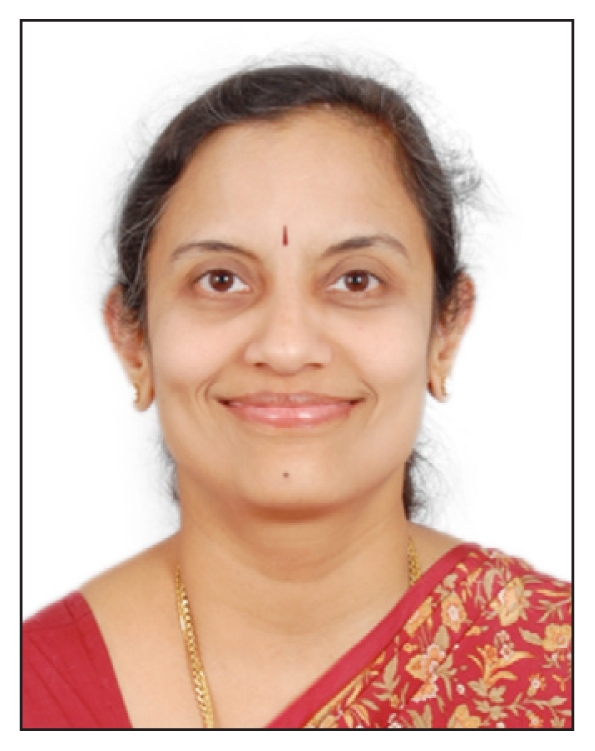
Kalpana Balakrishnan

**Figure:**
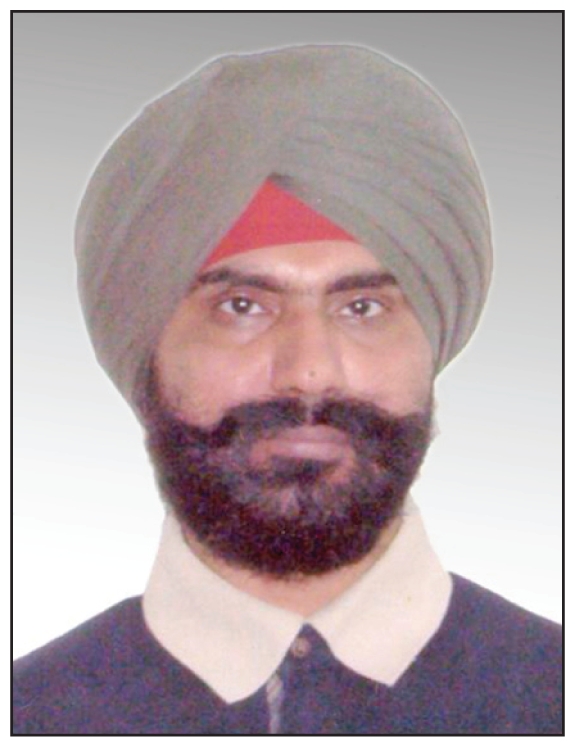
R.S. Dhaliwal

**Figure:**
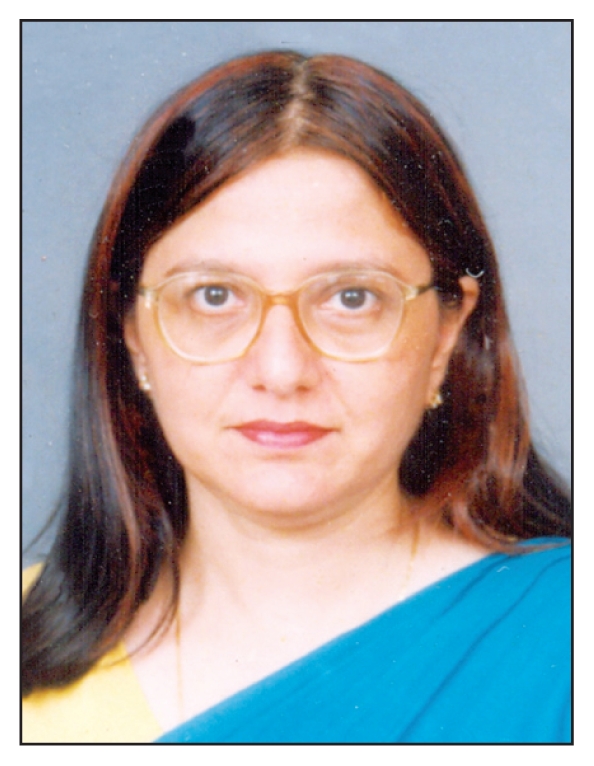
Bela Shah
